# Effects of Intimate Partner Violence During COVID-19 and Pandemic-Related Stress on the Mental and Physical Health of Women Veterans

**DOI:** 10.1007/s11606-022-07589-z

**Published:** 2022-08-30

**Authors:** Katherine M. Iverson, Christina M. Dardis, Sean Cowlishaw, Aliya R. Webermann, Danielle R. Shayani, Melissa E. Dichter, Karen S. Mitchell, Kristin M. Mattocks, Megan R. Gerber, Galina R. Portnoy

**Affiliations:** 1 Women’s Health Sciences Division of the National Center for PTSD, VA Boston Healthcare System, Boston, MA USA; 2 Department of Psychiatry, Boston University School of Medicine, Boston, MA USA; 3 Department of Psychology, Towson University, Towson, MD USA; 4 Phoenix Australia - Centre for Posttraumatic Mental Health, Department of Psychiatry, University of Melbourne, Carlton, VIC Australia; 5 VA Boston Healthcare System, Boston, MA USA; 6 VA Center for Health Equity Research and Promotion, Corporal Michael J. Crescenz VA Medical Center, Philadelphia, PA USA; 7 School of Social Work, Temple University, Philadelphia, PA USA; 8 VA Central Western Massachusetts Healthcare System, Leeds, MA USA; 9 Department of Quantitative Health Sciences, University of Massachusetts Medical School, Worcester, MA USA; 10 Division of General Medicine, Albany Medical College, Albany, NY USA; 11 Albany Stratton VA Medical Center, Albany, NY USA; 12 VA Connecticut Healthcare System, West Haven, CT USA; 13 Department of Psychiatry, Yale University, New Haven, CT USA

**Keywords:** cumulative stress, domestic violence, public health crises

## Abstract

**Background:**

Little is known about women veterans’ intimate partner violence (IPV) experiences during the COVID-19 pandemic or the impacts of pandemic-related stress on their mental and physical health.

**Objectives:**

To identify IPV experiences among women veterans prior to and during the pandemic, pandemic-related stressors, and examine their respective contributions to mental and physical health.

**Design:**

National sample of women veterans drawn from a larger web-based longitudinal study. Relationships between recent IPV and pandemic-related stressors were tested with linear regressions, controlling for pre-pandemic IPV and mental and physical health symptoms, demographic, and military-related covariates.

**Participants:**

One hundred forty-two women veterans (*M*_age_=58.8 years).

**Main Measures:**

We assessed IPV (CTS-2), PTSD (PCL-5), depression (CESD), anxiety (DASS-A), physical health (PHQ-15), and physical health–related quality of life (SF-12) prior to the pandemic (June 2016–December 2016/January 2017) and during the pandemic study period (March 2020–December 2020/January 2021). We assessed pandemic-related stressors (EPII) during the pandemic study period.

**Key Results:**

Over a third (38.7%) of participants experienced IPV during the pandemic study period (psychological: 35.9%, physical: 9.9%, sexual: 4.2%). Overall rates, frequency, and severity of IPV experience did not significantly differ between the pre-pandemic and pandemic study periods. Few participants tested positive for COVID-19 (4.2%); however, most participants reported experiencing pandemic-related stressors across life domains (e.g., social activities: 88%, physical health: 80.3%, emotional health: 68.3%). IPV during the pandemic and pandemic-related stressors were both associated with greater PTSD and depressive symptoms. Pandemic-related stressors were associated with worse anxiety and physical health symptoms. Neither IPV during the pandemic nor pandemic-related stressors were associated with physical health–related quality of life.

**Conclusions:**

IPV experiences during the pandemic were common among women veterans, as were pandemic-related stressors. Although IPV did not increase in the context of COVID-19, IPV experiences during the pandemic and pandemic-related stressors were linked with poorer mental and physical health.

There is global concern that the coronavirus disease 2019 (COVID-19) and associated lockdowns have increased the prevalence of intimate partner violence (IPV) among women and exacerbated IPV-related health impacts, including injury, depression, and posttraumatic stress disorder (PTSD).^1,2^ Pandemic stressors, including job loss, quarantining and social isolation, economic concerns, and relationship stress, are factors that may heighten risk for IPV^3,4^ and contribute to the pandemic’s negative impacts on individuals’ well-being.^5–8^

Estimates in the United States (U.S.) suggest that despite an overall decrease in the number of women seeking care for IPV, the incidence of physical IPV was 1.8 times greater during the pandemic relative to prior years and there was evidence of more severe IPV.^9^ Some studies from outside the U.S. have also found evidence of increased IPV,^10–13^ while another found evidence of a possible reduction in IPV.^14^ However, most of these studies have been cross-sectional and data was collected after the onset of COVID-19, introducing possible retrospective bias. Furthermore, some studies assess changes in calls to IPV hotlines^10,14^ or medical care for IPV^9^ rather than self-reported IPV, and may represent specific help-seeking samples. Prospective studies that include pre-pandemic measurement of self-reported IPV are needed to more accurately assess whether IPV has significantly increased during the pandemic.

Absent from research on IPV during the COVID-19 pandemic are women veterans, whom experience high risk for IPV.^15,16^ Women veterans also have greater trauma exposures than non-Veteran women.^17,18^ Military sexual trauma (MST) and combat exposure are prevalent military-related experiences among women veterans.^18,19^ Elevated trauma exposure likely exacerbates the potential risks of proximal COVID-19 stressors on physical and mental health outcomes, consistent with theories of cumulative stress and allostatic load.^20^

Little research has examined the impact of the COVID-19 pandemic on women veterans’ mental and physical health. Among pregnant veterans, Mattocks et al. found that mental health was the most frequently endorsed pandemic-related stressor (of eight categories; e.g., employment, childcare).^21^ Additionally, those with trauma histories, including combat exposure, MST, and/or IPV, were 2.5 times more likely to perceive COVID-19-related declines in their general mental health, which may reflect pre-existing trauma-related symptoms that were exacerbated by pandemic stress.^21^ This is consistent with findings from a cross-sectional survey of a predominantly male (95%) treatment-seeking sample of UK veterans that examined perceptions of personal mental health changes during COVID-19.^22^ Findings showed that depression, anxiety, and PTSD symptoms were commonly perceived to have been exacerbated by the pandemic and that experiencing more COVID-related stressors was associated with increased symptom severity.^22^ However, this study did not have pre-COVID comparison data, examine physical health, or control for MST or combat exposure, common determinants of mental and physical health among veterans.^23–25^

Given potential increases in IPV during the pandemic, the dearth of prospective research that includes pre-pandemic measurement of IPV and health-related constructs, and the paucity of literature on women veterans’ experiences of IPV and stressors during COVID-19, research is needed to examine IPV experiences, pandemic stressors, and physical and mental health among women veterans. This study aimed to investigate: (a) IPV experiences prior to and during the COVID-19 pandemic and (b) pandemic-related stressors experienced by women veterans. Additionally, we hypothesized that both (a) IPV experienced during the pandemic and (b) pandemic-related stressors would be uniquely associated with worse mental and physical health, when controlling for pre-pandemic IPV and mental and physical health.

## METHODS

### Participants and Procedures

This study utilized data from a larger national survey of women veterans conducted at four timepoints between November 2014 and January 2021. The original study was planned as a cross-sectional assessment of women’s preferences for IPV care,^26^ but was later extended to assess IPV and health at additional timepoints (described below). Participants completed web-based surveys administered by the Ipsos (formerly GfK) research firm. Ipsos maintains the KnowledgePanel®, a probability-based, non-volunteer access panel of 55,000 adults that is representative of 97% of U.S. households. Participants receive points for completing surveys that can be redeemed for prizes. For the initial survey, all women veterans in the KnowledgePanel® were eligible and received email invitations describing the purpose of the study (i.e., to understand women veterans’ health needs and counseling preferences related to unsafe or unhealthy intimate relationships) and other elements of informed consent. Women were informed they could participate regardless of whether they had experienced an unsafe or unhealthy relationship. Women received similar email invitations for the follow-up surveys, including the study’s purpose (i.e., to understand the impact of unsafe or unhealthy relationships on physical and mental health). Women were eligible for follow-up surveys if they had completed the prior survey and were still enrolled in the KnowledgePanel®. The VA Boston Healthcare System’s Institutional Review Board approved this study.

At the time of the initial 30-min survey (time 1), the KnowledgePanel® included 548 women veterans, 411 of whom participated (75% response rate). Subsequent 60-min follow-up surveys occurred at 18 months (time 2), 2 years (time 3), and 5 years (time 4) after time 1. At time 2, there were 330 eligible women, 266 of whom participated (81% response rate for eligible participants; 65% for time 1 participants). At time 3 (December 2016–January 2017), there were 261 eligible women and 190 participated (73% response rate for eligible participants; 71% for time 2 participants). At time 4 (December 2020–January 2021), there were 174 women eligible because they were still enrolled in the KnowledgePanel® and received an email invitation to participate in a follow-up study of women veterans’ relationship experiences and health needs during the COVID-19 pandemic, of whom 142 participated (82% response rate for eligible participants; 75% for time 3 participants). These 142 time 4 participants are the focus of this study. Women who participated in all four timepoints did not differ on time 1 demographic characteristics (i.e., age, race, education, income) or exposure to combat or military sexual trauma (MST) compared to women who did not complete all four timepoints. We refer to the time 4 timepoint as the “pandemic timepoint,” and compare this to the prior timepoint (time 3; “pre-pandemic”). Women who completed the pandemic timepoint did not differ from those who did not on any demographics (i.e., age, race, income, education, marital status), MST or combat exposure, or any time 3 IPV frequency or mental and physical health variables (*p*s > 0.05).

### Measures

Demographics, MST, and combat exposure were self-reported, as described previously.^26^

#### IPV

Physical, sexual, and psychological IPV were assessed at both timepoints using the Revised Conflict Tactics Scales (CTS-2).^27^ The pre-pandemic timepoint was anchored to “the past 6 months” and the pandemic timepoint was anchored to “since the beginning of the coronavirus pandemic in March this year.” Responses are on a 7-point scale (*never* to *more than 20 times*). We created dichotomous variables (+/−) and frequency variables for each IPV type and for each form of severe IPV.^27^ Total frequency scores represented overall IPV levels (*α* = 0.72 (pre-pandemic) and 0.74 (pandemic)).

#### Pandemic-Related Stressors

The Epidemic-Pandemic Impacts Inventory (EPII)^28^ was recently developed by stress and trauma measurement specialists via a process of expert consensus. The EPII assesses pandemic-related stressors across personal and social domains (e.g., work/employment, physical health, social activities, physical distancing and quarantine). Participants were asked to indicate whether the pandemic has impacted them personally (yes/no) across 73 items spanning 9 domains. All 9 domains included in this study index negative or stressful experiences.^29^ An exploratory latent class analysis of EPII items supports its utility as a measurement tool for assessing COVID-related stressful experiences.^29^ Items were summed for a total index of pandemic-related stressors (*α* = 0.90).

#### Mental and Physical Health Symptoms

The survey included mental and physical health measures that were assessed at the pre-pandemic and pandemic timepoints. *Depressive symptoms* were assessed with the 20-item Center for Epidemiological Studies-Depression Scale (CES-D).^30^ Higher scores reflect greater depressive symptoms (*α* = 0.92 (pre-pandemic) and 0.93 (pandemic)). *PTSD symptoms* were measured with the 20-item PTSD Checklist-5 (PCL-5).^31^ Responses were summed, with higher scores indicating greater symptoms (*α* = .97 at both timepoints). *Anxiety symptoms* were measured with the 14-item anxiety subscale of the Depression Anxiety Stress Scale (DASS-A).^32^ Higher sum scores reflect greater symptoms (*α* = 0.92 at both timepoints). *Physical health symptoms* were assessed with the 15-item Patient Health Questionnaire (PHQ-15).^33^ Higher sum scores indicate higher symptoms (*α* = 0.84 (pre-pandemic) and 0.85 (pandemic)). *Physical health–related quality-of-life* was measured using the Short-Form-12 Physical Component Score (SF-12 PCS).^34^ We summed the weighted means across domains; lower scores reflect worse functioning (*α* = 0.86 (pre-pandemic) and 0.87 (pandemic)).

### Data Analysis

We calculated descriptive statistics for IPV and mental and physical health variables at both timepoints, and frequencies of pandemic-related stressors. Paired-samples *t*-tests compared continuous variables and McNemar tests compared dichotomous variables; familywise error rates were controlled using the Benjamini-Hochberg method.^35^ After examining bivariate correlations among variables of interest, a series of linear regressions examined the associations between IPV during the pandemic and pandemic-related stress with mental and physical health, when controlling for pre-pandemic IPV and mental and physical health symptoms.^22,36,37^ Age, race/ethnicity, MST, and combat exposure were covariates as these factors are associated with mental and physical health outcomes among this population.^38–40^

## RESULTS

Table 1 displays the sample’s sociodemographic and military-related characteristics. Participants were an average of 58.8 years old and most (77.5%) identified as White/non-Hispanic. Nearly half of the sample reported MST (48%) and 11% reported combat exposure.

**Table 1:** Participant Characteristics (*N* = 142)

Variable	Mean (SD) or % (*N*)
Age, years	58.75 (13.16)
Race	
White, non-Hispanic	77.5% (110)
Non-White/Hispanic	22.5% (32)
Education	
< Bachelor’s degree	48.6% (69)
Bachelor’s degree or more	51.4% (73)
Employment status	
Employed	47.2% (67)
Unemployed/out of workforce	52.8% (75)
Income	
< $25,000	10.6% (15)
$25,000–$49,000	19.7% (28)
$50,000–$74,999	21.1% (30)
$75,000–$99,999	19.7% (28)
$100,000+	28.9% (41)
Marital status	
Married/cohabitating	67.6% (96)
Non-married/non-cohabitating	32.4% (46)
Sexual orientation	
Heterosexual/straight	90.1% (128)
Lesbian/gay	6.3% (9)
Bisexual	2.8% (4)
Did not answer	0.7% (1)
Military sexual trauma	
Yes	47.9% (68)
No	52.1% (74)
Combat exposure	
Yes	10.6% (15)
No	89.4% (127)

Note: Percentages may not equate to 100% due to rounding

Table 2 displays average levels and rates of any IPV and specific forms of IPV across the pre-pandemic and pandemic study periods. Overall, 38.7% of women reported any IPV during the pandemic study period (psychological: 35.9%, physical: 9.9%, and sexual: 4.2%). Additionally, 17.6% of women reported any severe IPV during the pandemic (severe psychological: 14.8%, severe physical: 4.2%, severe sexual: 1.4%). There were no significant differences between the two timepoints on any of the IPV variables, suggesting that women experienced similar rates, frequency, and severity of IPV during the pandemic study period as the pre-pandemic period. For example, as shown in Table 2, 47.2% of women experienced any IPV during the pre-pandemic period, and 38.7% reported any IPV during the pandemic period, a nonsignificant difference in proportions (*p=.*10). Specifically, examining the dichotomous variable representing any IPV, the majority of women reported stability in IPV experience across the timepoints (71.9%), by either reporting no IPV at both timepoints (42.3%; *n* = 60) or reporting IPV at both timepoints (29.6%; *n* = 42); however, 16.9% (*n* = 24) reported pre-pandemic IPV only and 9.2% (*n* = 13) reported IPV only during the pandemic. To explore demographic differences in IPV stability across the four groups, post hoc tests (using chi-square for categorical and ANOVA for continuous variables) revealed no significant differences in age, race/ethnicity, education, employment, income, or sexual orientation (*p*s > .05). Being married/cohabitating was significantly associated with group membership (omnibus *χ*^2^ (3) = 13.66, *p* = .003, *Φ*= .314), such that 88.1% of women who reported IPV at both timepoints were married/cohabitating, compared to 53.3% of women who reported no IPV at either timepoint (*p* <.05); women who experienced only pre-pandemic IPV (66.7%) and pandemic IPV only (69.2%) were intermediate to, and did not significantly differ from the other groups (*p*s >.05).

**Table 2:** Descriptive Statistics for Pre-pandemic and Pandemic-Related Variables of Interest (*N* = 142)

	Range	Pre-pandemic (June 2016–December 2016/January 2017)	Pandemic (March 2020–December 2020/January 2021)	
**Continuous**		***M (SD)***	***M (SD)***	***p***
Psychological IPV	0–74	5.29 (10.70)	4.54 (10.57)	0.32
Physical IPV	0–22	0.49 (2.37)	0.40 (2.15)	0.55
Sexual IPV	0–25	0.54 (2.70)	0.21 (1.10)	0.17
Total IPV	0–76	6.32 (12.78)	5.07 (11.87)	0.18
Severe psychological IPV	0–16	0.48 (1.43)	0.62 (2.14)	0.54
Severe physical IPV	0–15	0.09 (0.52)	0.22 (1.49)	0.30
Severe sexual IPV	0–4	0.04 (0.23)	0.05 (0.43)	0.81
Total severe IPV	0–28	0.61 (1.92)	0.89 (3.16)	0.33
PTSD symptoms (PCL-5)	0–68	10.46 (14.10)	10.28 (13.49)	0.87
Depressive symptoms (CESD)	0–46	12.02 (10.88)	11.84 (10.79)	0.70
Anxiety symptoms (DASS-A)	0–35	2.92 (5.00)	2.90 (4.98)	0.96
Physical health symptoms (PHQ-15)	0–22	6.76 (4.85)	6.77 (4.99)	0.96
**Physical health–related quality of life (SF-12)**	**22.58–63.98**	**47.84 (8.92)**	**45.62 (5.34)**	**0.001**
**Dichotomous IPV+ status**		**% (*n*)**	**% (*n*)**	***p***
Psychological IPV	--	46.5% (*n* = 66)	35.9% (*n* = 51)	0.03
Physical IPV	--	9.2% (*n* = 13)	9.9% (*n* = 14)	1.00
Sexual IPV	--	9.9% (*n* = 14)	4.2% (*n* = 6)	0.12
Any IPV	--	47.2% (*n* = 67)	38.7% (*n* = 55)	0.10
Severe psychological IPV	--	15.5% (*n* = 22)	14.8% (*n* = 21)	1.00
Severe physical IPV	--	4.2% (*n* = 6)	4.2% (*n* = 6)	1.00
Severe sexual IPV	--	3.5% (*n* = 5)	1.4% (*n* = 2)	0.38
Any severe IPV	--	15.5% (*n* = 22)	17.6% (*n* = 25)	0.66

Note: *p-*values represent the results of paired samples *t*-tests (continuous variables) or McNemar tests (dichotomous variables). Bolded results are significant following correction for family-wise error using the Benjamini-Hochberg procedure, with significant values indicating significant differences in the means (or proportions) of individuals who experienced IPV across the two timepoints and nonsignificant differences indicating stability in the means (or proportions) across the two timepoints.^35^ *IPV*, intimate partner violence; *PCL-5*, PTSD Checklist-5; *CESD*, Center for Epidemiologic Studies-Depression Scale; *DASS-A*, Anxiety Subscale of Depression Anxiety Stress Scale; *PHQ-15*, Patient Health Questionnaire-15; *SF-12*, Physical Component Score of the Short Form 12

Mental and physical health symptoms at the pre-pandemic and pandemic timepoints are reported in Table 2. Following correction for family-wise error, physical health–related quality of life decreased slightly between the two timepoints. None of the other health variables changed significantly between the timepoints (*p*s > 0.05).

In terms of pandemic-related stressors, nearly all women (98.6%) reported stressors from COVID-19 (Table 3). Whereas just a few participants tested positive for COVID-19 (4.2%), a range of negative effects on women’s lives, families, and careers were reported. Most participants (88.0%) reported their social activities were negatively impacted, and relatedly, 58.5% reported a need to physical distance and quarantine, including 30.3% who were isolated due to existing health conditions that increase the risk of COVID-19. Additionally, 80.3% reported physical health stressors (e.g., 66.2% were more sedentary, 47.2% were less physically active, and 43.0% overate or ate more unhealthy foods). The majority of women (68.3%) also reported pandemic-related stress in terms of emotional health and well-being, such as increases in mental health symptoms (31.0%), sleep problems (27.5%), and screen-time (59.2%). A plurality of women also reported pandemic-related stressors pertaining to their work (39.4%; e.g., increased workload) and home life (27.5%; e.g., caring for family).

**Table 3:** Frequencies of COVID-19 Pandemic–Related Stressors

	Frequency % (*n*)
**Work and employment**	**39.4% (*n*** **=56)**
1. Laid off from job or had to close own business	8.5% (*n* = 12)
2. Reduced work hours or furloughed	12.0% (*n* = 17)
3. Had to lay-off or furlough employees or people supervised	2.1% (*n* = 3)
4. Had to continue to work even though in close contact with people who might be infected	16.2% (*n* = 23)
5. Spend a lot of time disinfecting at home due to close contact with people who might be infected at work.	13.4% (*n* = 19)
6. Increase in workload or responsibilities	20.4% (*n* = 29)
7. Hard time doing job well because of needing to take care of people in the home.	4.2% (*n* = 6)
8. Hard time making the transition to working from home	7.0% (*n* = 10)
9. Provided direct care to people with the disease (e.g., doctor, nurse, patient care assistant, radiologist)	6.3% (*n* = 9)
10. Provided supportive care to people with the disease (e.g., medical support staff, custodial, administration)	4.9% (*n* = 7)
11. Provided care to people who died as a result of the disease	1.4% (*n* = 2)
**Education and training**	**18.3% (*n*** **= 26)**
12. Had a child in home who could not go to school	16.9% (*n* = 24)
13. Adult unable to go to school or training for weeks or had to withdraw	3.5% (*n* = 5)
**Home life**	**27.5% (*n*** **= 39)**
14. Childcare or babysitting unavailable when needed	4.2% (*n* = 6)
15. Difficulty taking care of children in the home	8.5% (*n* = 12)
16. More conflict with child or harsher disciplining of child	9.2% (*n* = 13)
17. Had to take over teaching or instructing a child	9.2% (*n* = 13)
18. Family or friends had to move into your home	2.1% (*n* = 3)
19. Had to spend a lot more time taking care of a family member	8.5% (*n* = 12)
20. Had to move or relocate	3.5% (*n* = 5)
21. Became homeless	0.7% (*n* = 1)
22. Increase in verbal arguments or conflict with a partner or spouse	13.4% (*n* = 19)
23. Increase in physical conflict with a partner or spouse	1.4% (*n* = 2)
24. Increase in verbal arguments or conflicts with other adult(s) in home	4.9% (*n* = 7)
25. Increase in physical conflict with other adult(s) in home	0.7% (*n* = 1)
26. Increase in physical conflict among children in home	2.1% (*n* = 3)
**Social activities**	**88.0% (*n*** **= 125)**
27. Separated from family or close friends	57.7% (*n* = 82)
28. Did not have the ability or resources to talk to family or friends while separated	11.3% (*n* = 16)
29. Unable to visit loved one in a care facility (e.g., nursing home, group home)	18.3% (*n* = 26)
30. Family celebrations cancelled or restricted	66.9% (*n* = 95)
31. Planned travel or vacations cancelled	69.7% (*n* = 99)
32. Religious or spiritual activities cancelled or restricted	51.4% (*n* = 73)
33. Unable to be with a close family member in critical condition	13.4% (*n* = 19)
34. Unable to attend in-person funeral or religious services for a family member or friend who died	24.6% (*n* = 35)
35. Unable to participate in social clubs, sports teams, or usual volunteer activities	48.6% (*n* = 69)
36. Unable to do enjoyable activities or hobbies	55.6% (*n* = 79)
**Economic**	**11.3% (*n*** **= 16)**
37. Unable to get enough food or healthy food	6.3% (*n* = 9)
38. Unable to access clean water	0% (*n* = 0)
39. Unable to pay important bills like rent or utilities	5.6% (*n* = 8)
40. Difficulty getting places due to less access to public transportation or concerns about safety	4.9% (*n* = 7)
41. Unable to get needed medications (e.g., prescriptions or over-the-counter)	2.8% (*n* = 4)
**Emotional health and well-being**	**68.3% (*n*** **= 97)**
42. Increase in child behavioral or emotional problems	9.9% (*n* = 14)
43. Increase in child’s sleep difficulties or nightmares	5.6% (*n* = 8)
44. Increase in mental health problems or symptoms (e.g., mood, anxiety, stress)	31.0% (*n* = 44)
45. Increase in sleep problems or poor sleep quality	27.5% (*n* = 39)
46. Increase in use of alcohol or substances	10.6% (*n* = 15)
47. Unable to access mental health treatment or therapy	3.5% (*n* = 5)
48. Not satisfied with changes in mental health treatment or therapy	6.3% (*n* = 9)
49. Spent more time on screens and devices (e.g., looking at phone, playing video games, watching TV)	59.2% (*n* = 84)
**Physical health problems**	**80.3% (*n*** **= 114)**
50. Increase in health problems not related to this disease	16.2% (*n* = 23)
51. Less physical activity or exercise	47.2% (*n* = 67)
52. Overeating or eating more unhealthy foods (e.g., junk food)	43.0% (*n* = 61)
53. More time sitting down or being sedentary	66.2% (*n* = 94)
54. Important medical procedure cancelled (e.g., surgery)	12.0% (*n* = 17)
55. Unable to access medical care for a serious condition (e.g., dialysis, chemotherapy)	0% (*n* = 0)
56. Got less medical care than usual (e.g., routine or preventive care appointments)	39.4% (*n* = 56)
57. Elderly or disabled family member not in the home unable to get the help they need	2.8% (*n* = 4)
**Physical distancing and quarantine**	**58.5% (*n*** **= 83)**
58. Isolated or quarantined due to possible exposure to this disease	30.3% (*n* = 43)
59. Isolated or quarantined due to symptoms or this disease	13.4% (*n* = 19)
60. Isolated due to existing health conditions the increase risk of infection or disease	30.3% (*n* = 43)
61. Limited physical closeness with child or loved one due to concerns of infection	32.4% (*n* = 46)
62. Moved out or lived away from family due to a high-risk job (e.g., healthcare worker, first responder)	0% (*n* =0)
63. Close family member not in the home was quarantined	7.7% (*n* = 11)
64. Family member was unable to return home due to quarantine or travel restrictions	2.8% (*n* = 4)
65. Entire household was quarantined for a week or longer	12.7% (*n* = 18)
**Infection history**	**16.9% (*n*** **= 24)**
66. Currently have symptoms of this disease but have not been tested	0.7% (*n* = 1)
67. Tested and currently have this disease	0% (*n* = 0)
68. Had symptoms of this disease but never tested	4.2% (*n* = 6)
69. Tested positive for this disease but no longer have it	4.2% (*n* = 6)
70. Got medical treatment due to severe symptoms of this disease	2.1% (*n* = 3)
71. Hospital stay due to this disease	0.7% (*n* = 1)
72. Someone died of this disease while in our home	0.7% (*n* = 1)
73. Death of close friend or family member from this disease	8.5% (*n* = 12)
**Any pandemic-related stressor**	**98.6% (*n*** **=140)**

As shown in Table 4, IPV during the pandemic was moderately and positively correlated with each of the mental and physical health variable (*r*s= 0.34–0.48), with the exception of physical health–related quality of life (*r*= −0.13). Pandemic stressors were moderately and positively associated with mental and physical health symptoms (*r*s= 0.44–0.57), and moderately and negatively correlated with physical health–related quality of life (*r*= −0.39). More pandemic stressors were associated with more IPV during the pandemic (*r*= 0.33).

**Table 4:** Correlation Matrix of Study Variables During the Pre-pandemic and Pandemic Study Periods

Variable	2	3	4	5	6	7	8	9	10	11	12	13
1. Pre IPV	0.40*	0.28*	0.32*	0.27*	-0.11	0.35*	0.53*	0.39*	0.31*	0.42*	0.24*	-0.20*
2.Pre PCL-5	-	0.76*	0.72*	0.68*	-0.39*	0.50*	0.32*	0.72*	0.62*	0.68*	0.54*	-0.35*
3. Pre CESD		-	0.68*	0.64*	-0.34*	0.44*	0.33*	0.64*	0.76*	0.64*	0.57*	-0.31*
4. Pre DASS-A			-	0.64*	-0.47*	0.37*	0.28*	0.56*	0.55*	0.68*	0.41*	-0.38*
5. Pre PHQ-15				-	-0.58*	0.43*	0.36*	0.60*	0.59*	0.61*	0.70*	-0.50*
6. Pre SF-12 PCS					-	-0.21*	-0.15	-0.35*	-0.34*	-0.40*	-0.41*	0.56*
7. Pandemic Stressors						-	0.33*	0.57*	0.51*	0.45*	0.44*	-0.24*
8. Pandemic IPV							-	0.48*	0.44*	0.39*	0.34*	-0.13
9. Pandemic PCL-5								-	0.80*	0.74*	0.64*	-0.37*
10. Pandemic CESD									-	0.72*	0.69*	-0.39*
11. Pandemic DASS-A										-	0.63*	-0.46*
12.Pandemic PHQ-15											-	-0.49*
13. Pandemic SF-12 PCS												-

Note: *p <.05. *Pre*, pre-pandemic study period (June 2016–December 2016/January 2017); *Pandemic*, pandemic study period (March 2020–December 2020/January 2021); *IPV*, intimate partner violence; *PCL-5*, PTSD Checklist-5; *CESD*, Center for Epidemiologic Studies-Depression Scale; *DASS-A*, Anxiety Subscale of Depression Anxiety Stress Scale; *PHQ-15*, Patient Health Questionnaire-15; *SF-12 PCS*, Physical Component Score of the Short Form 12; *Pandemic Stressors*, total pandemic-related stressors reported on the Epidemic-Pandemic Impacts Inventory

A series of linear regressions examined whether IPV during the pandemic and pandemic-related stressors were associated with worse mental and physical health (Table 5). As expected, higher levels of IPV experienced during the pandemic (*p* = 0.001) and pandemic-related stressors (*p* <0.001) were each uniquely associated with greater PTSD symptoms, with small effect sizes (*f*^2^s = 0.04 and 0.02, respectively). The same pattern of results was found for depressive symptoms (*f*^2^s = 0.03 and 0.02, respectively). Pandemic-related stressors were also associated with greater anxiety (*p* = 0.02, *f*^2^ = 0.02) and physical health symptoms (*p* = 0.04, *f*^2^ = 0.02), demonstrating small effects. Neither variable was associated with physical health–related quality of life.

**Table 5:** Linear Regressions Predicting Current Mental and Physical Health from IPV During the Pandemic and COVID-19 Pandemic–Related Stressors, While Controlling for Demographic Covariates, Military Sexual Trauma, Combat Exposure, and Pre-pandemic IPV Experiences and Pre-pandemic Mental and Physical Health Symptoms

	*β*	*t*	*p*	*f*^*2*^
*Pandemic PTSD symptoms (PCL-5)* (model statistics: *F* (8,136) = 30.36, *p* <0.001, *R*^2^ = 0.63)				
Pre-pandemic IPV experiences	−0.07	−1.02	0.31	0.003
**Pre-pandemic PTSD symptoms**	**0.54**	**8.37**	**<0.001**	**0.23**
**Pandemic IPV experiences**	**0.24**	**3.86**	**<0.001**	**0.04**
**Pandemic-related stressors**	**0.21**	**3.41**	**0.001**	**0.03**
*Pandemic depressive symptoms (CESD)* (model statistics: *F* (8,136) = 29.49, *p* <0.001, *R*^2^ = 0.63)				
Pre-pandemic IPV experiences	−0.03	−0.47	0.64	0.001
**Pre-pandemic depressive symptoms**	**0.63**	**10.23**	**<0.001**	**0.40**
**Pandemic IPV experiences**	**0.19**	**2.95**	**0.004**	**0.03**
**Pandemic-related stressors**	**0.18**	**2.92**	**0.004**	**0.02**
*Pandemic anxiety symptoms (DASS-A)* (model statistics: *F* (8,137) = 19.59, *p* <0.001, *R*^2^ = 0.52)				
Pre-pandemic IPV experiences	0.12	1.65	0.10	0.009
**Pre-pandemic anxiety symptoms**	**0.54**	**7.76**	**<0.001**	**0.27**
Pandemic IPV experiences	0.11	1.56	0.12	0.009
**Pandemic-related stressors**	**0.16**	**2.35**	**0.02**	**0.02**
*Pandemic physical health symptoms (PHQ-15)* (model statistics: *F* (8,137) = 18.84, *p* <0.001, *R*^2^ = 0.51)				
Pre-pandemic IPV experiences	−0.03	−0.38	0.70	0.001
**Pre-pandemic physical health symptoms**	**0.61**	**8.27**	**<0.001**	**0.33**
Pandemic IPV experiences	0.07	0.96	0.34	0.004
**Pandemic-related stressors**	**0.14**	**2.08**	**0.04**	**0.02**
*Pandemic physical health–related quality of life (SF-12 PCS)* (model statistics: *F* (8,133) = 7.67, *p* < 0.001, *R*^2^ = 0.29)				
Pre-pandemic IPV experiences	−0.14	−1.56	0.12	0.01
**Pre-pandemic SF-12 physical symptoms**	**0.52**	**6.58**	**<0.001**	**0.30**
Pandemic IPV experiences	−0.01	−0.09	0.93	<0.001
Pandemic-related stressors	−0.07	−0.81	0.42	0.003

Note: Covariates include age, race/ethnicity, combat exposure, and history of military sexual trauma. *Pre*, pre-pandemic timepoint (June 2016–December 2016/January 2017); *Pandemic*, pandemic period (December 2020/January 2021); *IPV*, intimate partner violence; *PCL-5*, PTSD Checklist-5; *CESD*, Center for Epidemiologic Studies-Depression Scale; *DASS-A*, Anxiety Subscale of Depression Anxiety Stress Scale; *PHQ-15*, Patient Health Questionnaire-15; *SF-12 PCS*, Physical Component Score of the Short Form 12; *COVID-19 Stressors*, total negative pandemic-related impacts reported on the Epidemic-Pandemic Impacts Inventory (EPII)

## DISCUSSION

The COVID-19 pandemic has impacted the life and functioning of individuals and communities worldwide, but little is known about stressors experienced among women veterans during COVID-19. This study contributes to the literature by being the first to examine IPV experiences both before and during the pandemic, as well as the implications of pandemic-related stressors for the mental and physical health of women veterans. Over one in three participants reported experiencing IPV during the first 9 to 10 months of the pandemic. The rates of IPV reported during the pandemic period were similar to those reported in this sample prior to the pandemic, but they are substantially higher than past-year rates in the general U.S. population during non-pandemic times.^41^ Although IPV experiences during the pandemic were not exacerbated in terms of overall rates, frequency, or severity of violence, they continued to remain high for this sample of women veterans. Most women (71.9%) reported stability in whether they did or did not experience IPV across time. While a small proportion of women reported IPV during, but not prior to, the pandemic (9.2%), there were no demographic characteristics (e.g., age, marital/cohabitating status) that differentiated this group. Additional research is needed to explore factors that might contribute to new IPV experiences during the pandemic.

IPV experiences during the pandemic negatively impacted women veterans’ PTSD and depressive symptoms. Additional research is needed to determine factors contributing to the IPV-related increases in mental health symptoms in the context of unchanged rates, frequency, and severity of IPV. Prior to COVID-19, women likely had more opportunities to access health services and social support to buffer against IPV’s emotional consequences,^42,43^ but the public health directives needed to manage COVID-19 (e.g., social distancing) could have resulted in increased isolation and reduced support, thereby increasing IPV’s impact on mental health during the pandemic.

Indeed, nearly all women reported pandemic-related stressors pertaining to their personal and social lives, with social activities being the most commonly endorsed category of stressors (88%), including reduced contact with loved ones and disruptions in spiritual practices. The stressor domain of physical health was the next most common (80%), followed by emotional health and well-being domain (68%). Our findings extend prior research with pregnant veterans^21^ by documenting a wider range of pandemic-related stressors experienced by women veterans and by examining the impacts of pandemic-related stress on women’s mental and physical health. Findings also extend prior research demonstrating that COVID-19 stress was linked with UK veterans’ *perceptions of* worse mental health during the pandemic.^22^ However, that study included only a small fraction of women (5%) and did not adjust for military stressors that are common determinants of mental and physical health among veterans.^23–25^ When adjusting for combat and MST exposure, we found a “dose-response” relationship between increasing numbers of pandemic-related stressors and increasing symptom severity across PTSD, depression, and anxiety. Our findings further extend the dose-response relationship to physical health problems.

In the context of the COVID-19 pandemic, there are many potential mechanisms by which pandemic stressors could negatively impact mental and physical health, particularly declines in social connection caused by social distancing and quarantining. Social connection helps people cope with stress while a lack of connectedness can increase psychological distress.^44^ Furthermore, the chronic stress from the pandemic can be expressed somatically and the associations between pandemic stressors and physical health symptoms observed in this study may reflect women’s physiological response to the chronic and wide-ranging stress of the pandemic.^20,45^

Contrary to our hypothesis, neither IPV experienced during the pandemic nor COVID-19 stressors were associated with physical health–related quality of life in multivariate analyses, though there was an overall decrease in physical health functioning since the pre-pandemic study period. The lack of association was surprising given prior research linking IPV experience to poorer physical health functioning above and beyond the effects of military stressors among post-9/11 veterans^46^ and the current samples’ high endorsement of stressors related to lack of physical health maintenance (i.e., two-thirds of the sample reported being more sedentary and nearly half reported getting less exercise). More research is needed to understand how these factors relate to one another and impact women veterans’ physical health–related quality of life.

This study has limitations that can be addressed in future studies. First, this is not a nationally representative sample. There may be ways in which the study participants differ from the broader women Veteran population. For example, 23% of the sample were racial minorities compared to 29% of the woman veteran population^47^ and the average participant was in her late-fifties. Although women ages 55–64 comprise the largest age group of women veterans,^47^ findings may not generalize to younger or more diverse women veterans. Similarly, half of the sample had a Bachelor’s degree or higher compared to 40% of the women veteran population yet the sample’s income was similar to the broader women veteran population.^48^ Future research would benefit from larger, nationally representative samples. Additionally, there was a 4-year interval between the pre-pandemic and pandemic timepoints and the IPV assessments were anchored to different timeframes. Given that the pandemic timeframe was 1.5 times longer than the pre-pandemic timeframe yet the IPV reports are similar across timepoints, it is possible that the findings may reflect a decrease in IPV. Longitudinal research designs incorporating three or more timepoints and consistent timeframes for assessing IPV are needed to confirm the temporal relationships of the variables examined in this study. Unfortunately, the prior assessments for the larger study do not include consistent measurements and timeframes for assessing IPV. Further research should examine factors that may mitigate any long-term impacts of these experiences on mental and physical health (e.g., social connectedness, stress management, and coping skills) to enable a better understanding of how these can be harnessed to promote further resiliency against IPV-related distress and effective management of pandemic-related stress.

There has been large increases in the number of women veterans using the Vetearns Health Administration (VHA) in recent years^40^, yet many still seek care at non-VHA facilities,^49^ underscoring the need for both VHA and non-VHA providers to understand the IPV and pandemic-related mental and physical health needs of women veterans. Our findings reinforce the importance of screening women for IPV and highlight the potential value of inquiring about pandemic-related stressors and offering appropriate interventions. Being aware of patients’ recent experiences with IPV and pandemic stressors, while understanding their connections with mental and physical health, may enable providers to better coordinate care and aid in treatment planning.

These findings have implications for VHA, which strives to ensure women’s mental and physical health needs pertaining to IPV and stress are addressed amidst the pandemic and beyond.^50^ In-person and telehealth services can include trauma-informed assessment of IPV and pandemic-related stress and providing physical and emotional risk assessment, safety planning, stress management, primary care, and mental health services. Evidence-based psychotherapies that are commonly available within VHA can reduce mental health symptoms from IPV *and* other stressors while also reducing risk for future IPV.^51^ Some VHA facilities are implementing a skills-based counseling intervention for IPV that is effective in improving women’s self-efficacy, empowerment, and mental health.^52,53^ Such interventions can address IPV and potentially increase self-efficacy to manage additional stress from the pandemic.

Although this sample of women veterans did not experience increased IPV in the context of COVID-19, women continued to experience high rates of IPV and additionally reported substantial pandemic-related stress, both of which contributed to poorer mental and physical health. This study underscores the importance of ensuring access to healthcare services for women veterans and can inform intervention strategies by encouraging researchers to elucidate ways in which women cope with violent relationships and other stressors during the COVID-19 pandemic and other public health crises.

## References

[1] **Sabri B, Hartley M, Saha J, Murray S, Glass N, Campbell JC.** Effect of COVID-19 pandemic on women’s health and safety: a study of immigrant survivors of intimate partner violence. Health Care Women Int. 2020;41(11-12):1-19. 10.1080/07399332.2020.1833012 PMC7902436 33085577

[2] **Sharma A, Borah SB.** Covid-19 and domestic violence: an indirect path to social and economic crisis. Journal Fam Violence. 2020:1-7. 10.1007/s10896-020-00188-8 PMC7386835 32836737

[3] Kaukinen C (2020). When stay-at-home orders leave victims unsafe at home: Exploring the risk and consequences of intimate partner violence during the COVID-19 pandemic. J Crim Justice..

[4] Viero A, Barbara G, Montisci M, Kustermann K, Cattaneo C (2020). Violence against women in the Covid-19 pandemic: a review of the literature and a call for shared strategies to tackle health and social emergencies. Forensic Sci Int..

[5] Pfefferbaum B, North CS (2020). Mental health and the Covid-19 pandemic. N Engl J Med..

[6] **Qiu J, Shen B, Zhao M, Wang Z, Xie B, Xu Y.** A nationwide survey of psychological distress among Chinese people in the COVID-19 epidemic: implications and policy recommendations. Gen Psychiatry. 2020;33(2). 10.1136/gpsych-2020-100213 PMC7061893 32215365

[7] Caparros-Gonzalez RA, Alderdice F (2020). The COVID-19 pandemic and perinatal mental health. J Reprod Infant Psychol..

[8] **Tadesse AW, Tarekegn SM, Wagaw GB, Muluneh MD, Kassa AM.** Prevalence and associated factors of intimate partner violence among married women during CoViD-19 pandemic restrictions: a community-based study. J Interpers Violence. 2020:0886260520976222. 10.1177/0886260520976222 PMC9160437 33289437

[9] Gosangi B, Park H, Thomas R (2021). Exacerbation of physical intimate partner violence during COVID-19 pandemic. Radiology..

[10] Agüero JM (2021). COVID-19 and the rise of intimate partner violence. World development..

[11] Aolymat I (2020). A cross-sectional study of the impact of CoViD-19 on domestic violence, menstruation, genital tract health, and contraception use among women in Jordan. The American journal of tropical medicine and hygiene..

[12] Hamadani JD, Hasan MI, Baldi AJ (2020). Immediate impact of stay-at-home orders to control COVID-19 transmission on socioeconomic conditions, food insecurity, mental health, and intimate partner violence in Bangladeshi women and their families: an interrupted time series. The Lancet Global Health..

[13] Sediri S, Zgueb Y, Ouanes S (2020). Women’s mental health: acute impact of COVID-19 pandemic on domestic violence. Arch Womens Ment Health..

[14] Barbara G, Facchin F, Micci L (2020). COVID-19, lockdown, and intimate partner violence: some data from an Italian service and suggestions for future approaches. J Womens Health..

[15] Dichter ME, Cerulli C, Bossarte RM (2011). Intimate partner violence victimization among women veterans and associated heart health risks. Womens Health Issues..

[16] **Cowlishaw S, Freijah I, Kartal D, et al.** Intimate partner violence (IPV) in military and veteran populations: a systematic review and meta-analysis of population-based surveys and population screening studies. Under review. 10.3390/ijerph19148853 PMC9316917 35886702

[17] McCauley HL, Blosnich JR, Dichter ME (2015). Adverse childhood experiences and adult health outcomes among veteran and non-veteran women. J Womens Health..

[18] Zinzow HM, Grubaugh AL, Monnier J, Suffoletta-Maierle S, Frueh BC (2007). Trauma among female veterans: a critical review. Trauma Violence Abuse..

[19] Vogt D, Vaughn R, Glickman ME (2011). Gender differences in combat-related stressors and their association with postdeployment mental health in a nationally representative sample of US OEF/OIF veterans. J Abnorm Psychol..

[20] McEwen BS, Stellar E (1993). Stress and the individual: mechanisms leading to disease. Arch Intern Med..

[21] **Mattocks K, Kroll-Desrosiers A, Vance V, et al.** Veterans’ perinatal care and mental health experiences during the COVID-19 pandemic: an examination of the role of prior trauma and pandemic-related stress. J Women's Health. 2022. 10.1089/jwh.2021.0209 35230179

[22] **Murphy D, Williamson C, Baumann J, Busuttil W, Fear N.** Exploring the impact of COVID-19 and restrictions to daily living as a result of social distancing within veterans with pre-existing mental health difficulties. BMJ Mil Health. 2022;168(1):2-33. 10.1136/bmjmilitary-2020-001622 33243764

[23] Arditte Hall KA, Bartlett BA, Iverson KM, Mitchell KS (2018). Eating disorder symptoms in female veterans: the role of childhood, adult, and military trauma exposure. Psychol Trauma..

[24] Kimerling R, Street AE, Pavao J (2010). Military-related sexual trauma among Veterans Health Administration patients returning from Afghanistan and Iraq. Am J Public Health..

[25] Scott JC, Pietrzak RH, Southwick SM (2014). Military sexual trauma interacts with combat exposure to increase risk for posttraumatic stress symptomatology in female Iraq and Afghanistan veterans. J Clin Psychiatry..

[26] Iverson KM, Stirman SW, Street AE (2016). Female veterans' preferences for counseling related to intimate partner violence: informing patient-centered interventions. Gen Hosp Psychiatry..

[27] Straus MA, Hamby SL, Boney-McCoy S, Sugarman DB (1996). The revised conflict tactics scales (CTS2) development and preliminary psychometric data. J Fam Issues..

[28] **Grasso DJ, Briggs-Gowan MJ, Ford JD, Carter A.** The Epidemic–Pandemic Impacts Inventory (EPII). University of Connecticut School of Medicine. 2020.

[29] **Grasso DJ, Briggs-Gowan MJ, Carter AS, Goldstein B, Ford JD.** A person-centered approach to profiling COVID-related experiences in the United States: preliminary findings from the Epidemic-Pandemic Impacts Inventory (EPII). PsyArXiv. 2021. 10.1002/brb3.2197 PMC8413802 34216110

[30] Radloff LS (1977). The CES-D scale: a self-report depression scale for research in the general population. Appl Psychol Meas..

[31] **Weathers FW, Litz BT, Keane TM, Palmieri PA, Marx BP, Schnurr PP.** The PTSD Checklist for DSM-5 (pcl-5). *Scale* available from the National Center for PTSD at www ptsd va gov. 2013;10.

[32] Lovibond PF, Lovibond SH (1995). The structure of negative emotional states: comparison of the Depression Anxiety Stress Scales (DASS) with the Beck Depression and Anxiety Inventories. Behav Res Ther..

[33] Kroenke K, Spitzer RL, Williams JB (2002). The PHQ-15: validity of a new measure for evaluating the severity of somatic symptoms. Psychosom Med..

[34] **Ware Jr JE, Kosinski M, Keller SD.** A 12-Item Short-Form Health Survey: construction of scales and preliminary tests of reliability and validity. Med Care. 1996:220-233. 10.1097/00005650-199603000-00003 8628042

[35] Benjamini Y, Hochberg Y (1995). Controlling the false discovery rate: a practical and powerful approach to multiple testing. J R Stat Soc Series B Stat Methodol..

[36] Dardis CM, Dichter ME, Iverson KM (2018). Empowerment, PTSD and revictimization among women who have experienced intimate partner violence. Psychiatry Res..

[37] Portnoy GA, Relyea MR, Street AE, Haskell SG, Iverson KM (2020). A longitudinal analysis of women veterans’ partner violence perpetration: the roles of interpersonal trauma and posttraumatic stress symptoms. J Fam Violence..

[38] Booth BM, Davis TD, Cheney AM, Mengeling MA, Torner JC, Sadler AG (2012). Physical health status of female veterans: contributions of sex partnership and in-military rape. Psychosom Med..

[39] Smith BN, Tyzik AL, Iverson KM (2015). Age-related differences in trauma exposure, PTSD symptomatology, and functional health and well-being in women veterans. Traumatology (Tallahass Fla)..

[40] **Frayne S, Phibbs C, Saechao F, et al.** Sourcebook: Women Veterans in the Veterans Health Administration. Longitudinal Trends in Sociodemographics, Utilization, Health Profile, and Geographic Distribution Women’s Health Evaluation Initiative, Women’s Health Services, Veterans Health Administration. Washington DC. : Department of Veterans Affairs; February 2018.

[41] Black M, Basile K, Breiding M (2011). National intimate partner and sexual violence survey: 2010 summary report.

[42] Connor J, Madhavan S, Mokashi M (2020). Health risks and outcomes that disproportionately affect women during the Covid-19 pandemic: a review. Soc Sci Med..

[43] **Roesch E, Amin A, Gupta J, García-Moreno C.** Violence against women during covid-19 pandemic restrictions. BMJ. 2020. 10.1136/bmj.m1712 PMC7202944 32381644

[44] Van Bavel JJ, Baicker K, Boggio PS (2020). Using social and behavioural science to support COVID-19 pandemic response. Nat Hum Behav..

[45] Lazarus RS, Folkman S (1984). Stress, appraisal, and coping.

[46] Iverson KM, Vogt D, Maskin RM, Smith BN (2017). Intimate partner violence victimization and associated implications for health and functioning among male and female post-9/11 veterans. Med Care..

[47] **U.S. Department of Veterans Affairs Office of the Actuary.** Veteran Population Projection Model-VetPop2021.

[48] **U.S. Census Bureau.** Current Veteran Population Supplemental Survey; 2019.

[49] Washington DL, Farmer MM, Mor SS, Canning M, Yano EM (2015). Assessment of the healthcare needs and barriers to VA use experienced by women veterans: findings from the national survey of women Veterans. Med Care..

[50] **Rossi FS, Shankar M, Buckholdt K, Bailey Y, Israni ST, Iverson KM.** Trying times and trying out solutions: intimate partner violence screening and support for women veterans during COVID-19. J Gen Intern Med. 2020;35(9):2728-2731. 10.1007/s11606-020-05990-0 PMC7325833 32607932

[51] Iverson KM, Gradus JL, Resick PA, Suvak MK, Smith KF, Monson CM (2011). Cognitive–behavioral therapy for PTSD and depression symptoms reduces risk for future intimate partner violence among interpersonal trauma survivors. J Consult Clin Psychol..

[52] **Iverson KM, Danitz SB, Driscoll M, et al.** Recovering from intimate partner violence through strengths and empowerment (RISE): development, pilot testing, and refinement of a patient-centered brief counseling intervention for women. Psychol Serv. Advanced Online Publication. 10.1037/ser0000544 34110870

[53] **Iverson KM, Danitz SB, Shayani DR, et al.** Recovering from intimate partner violence through strengths and empowerment: findings from a randomized clinical trial. J Clin Psychiatry. 2022;83(1):38188. 10.4088/JCP.21m14041 34813687

